# The anti-cancer activity of an andrographolide analogue functions through a GSK-3β-independent Wnt/β-catenin signaling pathway in colorectal cancer cells

**DOI:** 10.1038/s41598-018-26278-8

**Published:** 2018-05-21

**Authors:** Somrudee Reabroi, Rungnapha Saeeng, Nittaya Boonmuen, Teerapich Kasemsuk, Witchuda Saengsawang, Kanoknetr Suksen, Weiming Zhu, Pawinee Piyachaturawat, Arthit Chairoungdua

**Affiliations:** 1 Department of Physiology, Faculty of Science, Mahidol University, Bangkok, 10400 Thailand; 2 Department of Chemistry and Center for Innovation in Chemistry, Faculty of Science, Burapha University, Chonburi, 20131 Thailand; 3 Key Laboratory of Marine Drugs, Ministry of Education of China, School of Medicine and Pharmacy, Ocean University of China, Qingdao, 266003 China; 4 Excellent Center for Drug Discovery (ECDD), Mahidol University, Bangkok, 10400 Thailand

## Abstract

The Wnt/β-catenin signaling pathway plays a key role in the progression of human colorectal cancers (CRCs) and is one of the leading targets of chemotherapy agents developed for CRC. The present study aimed to investigate the anti-cancer effects and molecular mechanisms of 19-*O*-triphenylmethyl andrographolide (RS-PP-050), an andrographolide analogue and determine its activity in the Wnt/β-catenin pathway. RS-PP-050 was found to potently inhibit the proliferation and survival of HT-29 CRC cells. It induces cell cycle arrest and promotes apoptotic cell death which was associated with the activation of PARP-1 and p53. Furthermore, RS-PP-050 exerts inhibitory effects on β-catenin transcription by suppressing T-cell factor/lymphocyte enhancer factor (TCF/LEF) activity in cells overexpressing β-catenin and by down-regulating the endogenous expression of Wnt target genes. RS-PP-050 also decreased the protein expression of the active form of β-catenin but functions independently of GSK-3β, a negative regulator of Wnt. Interestingly, RS-PP-050 extensively blocks phosphorylation at Ser675 of β-catenin which links to interference of the nuclear translocation of β-catenin and might contribute to Wnt inactivation. Collectively, our findings reveal the underlying anti-cancer mechanism of an andrographolide analogue and provide useful insight for exploiting a newly chemotherapeutic agent in Wnt/β-catenin-overexpressing CRC cells.

## Introduction

Colorectal cancer (CRC), is a malignancy that has one of the highest incidence rates in both males and females in the United States^1^. Although the early response success rate of CRC to current chemotherapeutic drugs is high, the invariable development of drug resistance as well as patient intolerance of adverse drug side effects limits their usage^2^. Thus, searching for new effective chemical entities with greater specificity is urgently needed. Advancement in cancer treatment strategies depend upon gaining a better understanding of the intracellular signaling cascades and molecular pathways involved in tumor survival and progression.

The deregulation of Wnt/β-catenin signaling is considered to be a major oncogenic event in the initiation and progression of most colon cancers^3^. In the absence of Wnt ligands, the key effector of this signaling, β-catenin, is sequestered in a destruction complex composed of adenomatous polyposis coli (APC), casein kinase 1α (CK1α), Axin, and glycogen synthase kinase-3β (GSK-3β) which maintains the activity of cytosolic β-catenin at a low level^4^. Ultimately, phosphorylation of the N-terminal β-catenin by GSK-3β at Ser33/Ser37/Thr41 residues marks β-catenin for degradation in the proteasome. In cancer cells, the binding of Wnt ligands to the Frizzled (Fz) receptor and the low density lipoprotein receptor-related protein 5/6 (LRP5/6) co-receptor on the plasma membrane triggers the disassembly of the destruction complex. This results in the accumulation of free β-catenin able to translocate into the nucleus to form a complex with TCF/LEF family transcription factors to activate the transcription of Wnt target genes such as c-myc, cyclin D1, matrix metalloproteinase-7 (MMP-7), and survivin. These target genes are responsible for colon tumor proliferation, survival and metastasis^5–8^.

The level of β-catenin is negatively controlled by canonical GSK-3β-mediated phosphorylation or other degradation pathways^9,10^. Under pathological conditions, however, loss or gain of function by genetic alterations of Wnt components including APC, Axin, and β-catenin, cause β-catenin to escape degradation imposed by the upstream negative regulators, allowing deregulated β-catenin transcriptional activity in the nucleus^11^. Because of these genetic mutations in the Wnt pathway components, inhibition upstream of β-catenin may produce the discernable effect to inactivate Wnt signaling. However, targeting events downstream of β-catenin towards the nuclear vicinity also offers a much more fascinating option for efficiently suppressing Wnt/β-catenin transactivation^12^. Recently, the association of protein kinase A (PKA) signaling has been implicated in positively influencing nuclear β-catenin levels and the transcriptional activity of Wnt signaling. Specific phosphorylation on Ser552 or Ser675 on the C-terminus of β-catenin by PKA leads to an increase in nuclear translocation of β-catenin and subsequently enhanced TCF/ LEF transcriptional activity in many cell types^13,14^. Aberrant accumulation of β-catenin in nucleus caused by Wnt gene mutations in CRC is thus a potential target to search for specific chemotherapeutic agents^15,16^.

Natural products have been a major source of therapeutic agents for the pharmaceutical industry^17^. A number of anti-cancer drugs originating from natural products are currently used in the clinic for treatment of CRC patients. Of these, andrographolide, a plant-based compound, that is a major constituent of *Andrographis paniculata* has shown great promise as an anti-cancer compound against a number of cancer cell types^18,19^. A recent study demonstrated that andrographolide promoted cell death through endoplasmic reticulum (ER) stress-mediated apoptosis through the induction of intracellular ROS generation and caused the activation of proapoptosis signaling, cell cycle arrest and inhibition of cellular pathways related to cancer cell survival^20^. However, it has not been used as a chemotherapeutic agent in clinic due to its low potency and poor oral bioavailability^21,22^. To overcome these limitations, chemical transformation to obtain analogues superior to the parent andrographolide has been an area of active research^23–25^. In our earlier study, a number of andrographolide analogues were developed, which showed interesting cytotoxicity profiles against a panel of cancer cell lines, acting through yet to be determined mechanisms of action^26,27^. The present study aimed to investigate the potent anti-cancer activity of 19-*O*-triphenylmethyl andrographolide (RS-PP-050), an andrographolide analogue, against CRC cells. The effects of the compound on the Wnt signaling pathway was explored. Here, we report that the anti-cancer activity of RS-PP-050 involves the inhibition of Wnt/β-catenin signaling via a GSK-3β-independent pathway. This is the first study which demonstrates the anti-cancer activity of the andrographolide analogue (RS-PP-050) which may hold great therapeutic potential for targeting Wnt/β-catenin-overactivated cancer cells.

## Results

### Inhibition of proliferation and survival of colorectal (CRC) cells by RS-PP-050

**Figure 1:**
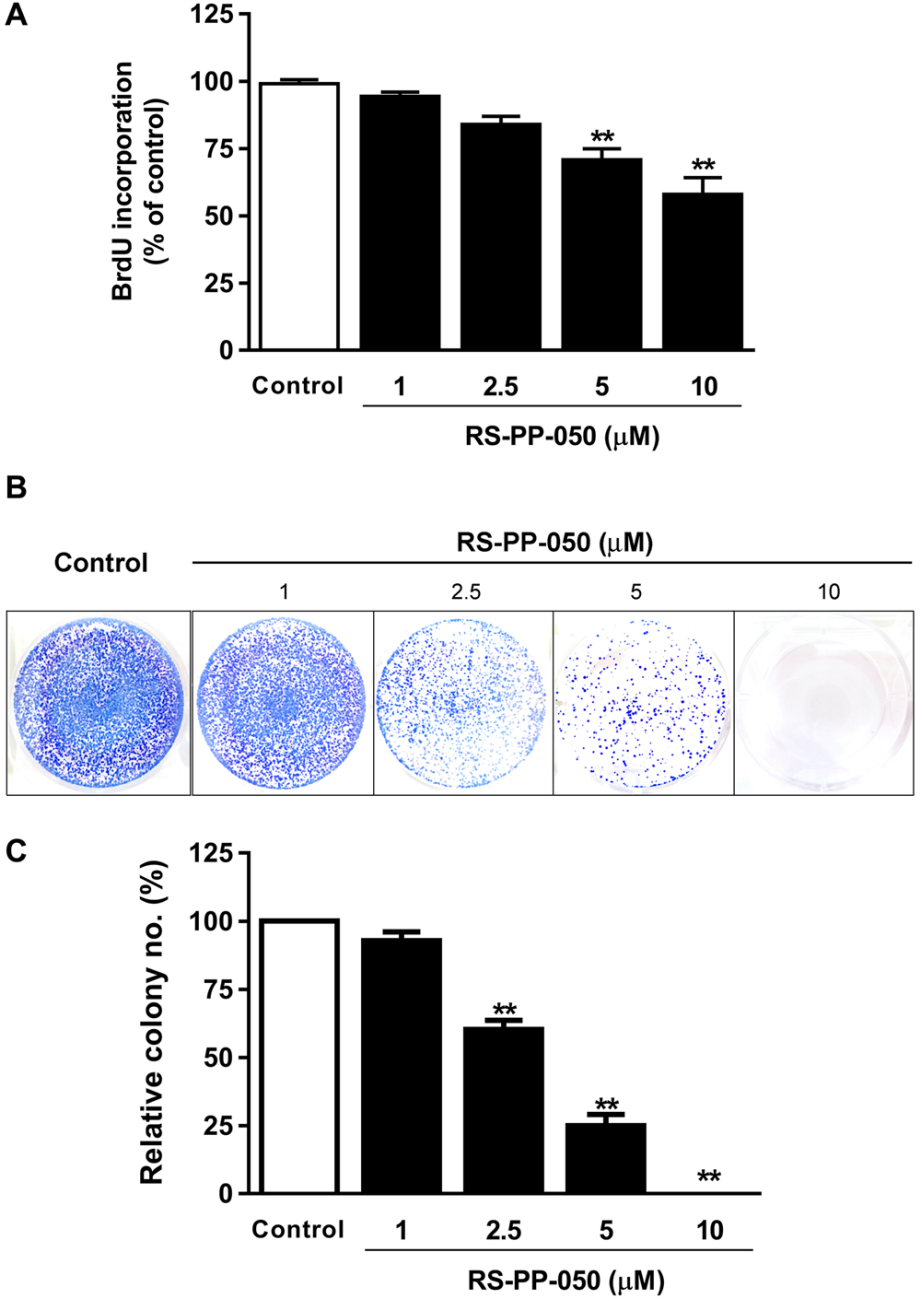
Inhibition of proliferation and survival of colorectal cancer (CRC) cells by RS-PP-050. (**A**) The anti-proliferative effect on HT-29 cells determined by BrdU incorporation after treatment with RS-PP-050 for 24 h. Data are means ± S.E.M compared with a vehicle control (0.1% DMSO) (n = 3) and presented as % of cell proliferation (**P < 0.01). (**B**) The inhibitory effect on HT-29 colony formation that was determined by a clonogenic assay upon treatment with RS-PP-050 for 15 days. (**C**) Bar graph showing the quantification data for the relative numbers of HT-29 colonies. Data are means ± S.E.M compared with the vehicle control (n = 3) and presented as % of relative colony numbers (**P < 0.01).

The cytotoxic effect of the andrographolide analogue, RS-PP-050, was investigated in three human CRC cell lines; HT-29, HCT116, and SW480 using an MTT viability assay. Vinblastine, doxorubicin and ellipticine were used as positive controls. Cells were treated with RS-PP-050 at various concentrations for 24, 48 or 72 h. Among the three CRC cell lines, HT-29 was the most sensitive cell line to the analogue, with an IC_50_ value of 11.43 ± 1.52 μM after 24 h of treatment, whereas those of SW480, and HCT116 cells were 15.88 ± 3.32 μM and 18.72 ± 1.83 μM, respectively (Table 1). IC_50_ value of RS-PP-050 on Chang liver cells (human normal liver cells) was higher than 100 µM. This indicates a lower cytotoxicity of RS-PP-050 to normal cells. IC_50_ values of RS-PP-050, andrographolide and the positive controls at various time points in HT-29 cells are shown in Table 2. RS-PP-050 decreased the cellular viability in a dose, and time dependent manner. It was more potent than the parent compound, andrographolide (IC_50_ = 48.64 ± 3.25 μM at 24 h). Based on the results, HT-29 cells were used for further mechanistic investigations with concentrations of RS-PP-050 ranging from 1–10 µM. RS-PP-050 was found to suppress the cellular proliferation of HT-29 in dose-dependent manner using a BrdU incorporation assay (Fig. 1A). To measure cell survival during long-term treatment with RS-PP-050, a clonogenic assay was performed. It was found that RS-PP-050 inhibited the ability of HT-29 cells to form colonies after 15 days of culture in a dose-dependent manner (Fig. 1B,C). A few colony numbers were found for treatment with 5 μM of RS-PP-050, while no colonies were observed at 10 μM RS-PP-050 after treatment. Taken together, these data indicate the potent anti-cancer effects of RS-PP-050 on CRC cells.

**Table 1:** IC_50_ values (µM) for RS-PP-050 in three CRC cell lines at various time points.

CRC cell lines	IC_50_ (μM) for RS-PP-050		
	24 h	48 h	72 h
HT-29	11.43 ± 1.52	3.41 ± 0.82	3.25 ± 0.81
HCT116	18.72 ± 1.83	3.94 ± 0.58	3.15 ± 0.46
SW480	15.88 ± 3.32	3.12 ± 0.10	2.90 ± 0.06

IC_50_ values (µM) of three CRC cell lines (HT-29, HCT116, and SW480) after treatment with RS-PP-050 for 24, 48, and 72 h. Cell viability was assessed with MTT assay. IC_50_ value is the concentration that inhibits 50% of cell proliferation. Each value represents as mean ± SEM compared to vehicle control from three independent experiments repeated in triplicates.

**Table 2:** IC_50_ values (µM) for the tested compounds in HT-29 cells at various time points.

Compounds	Time (h)		
	24	48	72
RS-PP-050	11.43 ± 1.52	3.41 ± 0.82	3.25 ± 0.81
Andrographolide	48.64 ± 3.25	31.79 ± 3.14	25.64 ± 3.03
Vinblastine	5.02 ± 1.02	0.07 ± 0.03	0.05 ± 0.02
Doxorubicin	25.90 ± 0.07	3.29 ± 0.49	1.29 ± 0.79
Ellipticine	13.35 ± 1.11	8.18 ± 1.89	4.66 ± 1.19

IC_50_ values (µM) of HT-29 cells after treatment with the tested compounds; RS-PP-050, andrographolide, vinblastine, doxorubicin, and ellipticine for 24, 48, and 72 h. Cell viability was assessed with MTT assay. IC_50_ value is the concentration that inhibits 50% of cell proliferation. Each value represents as mean ± SEM compared to the vehicle control from three independent experiments repeated in triplicates.

### Induction of apoptosis and cell cycle arrest by RS-PP-050

**Figure 2:**
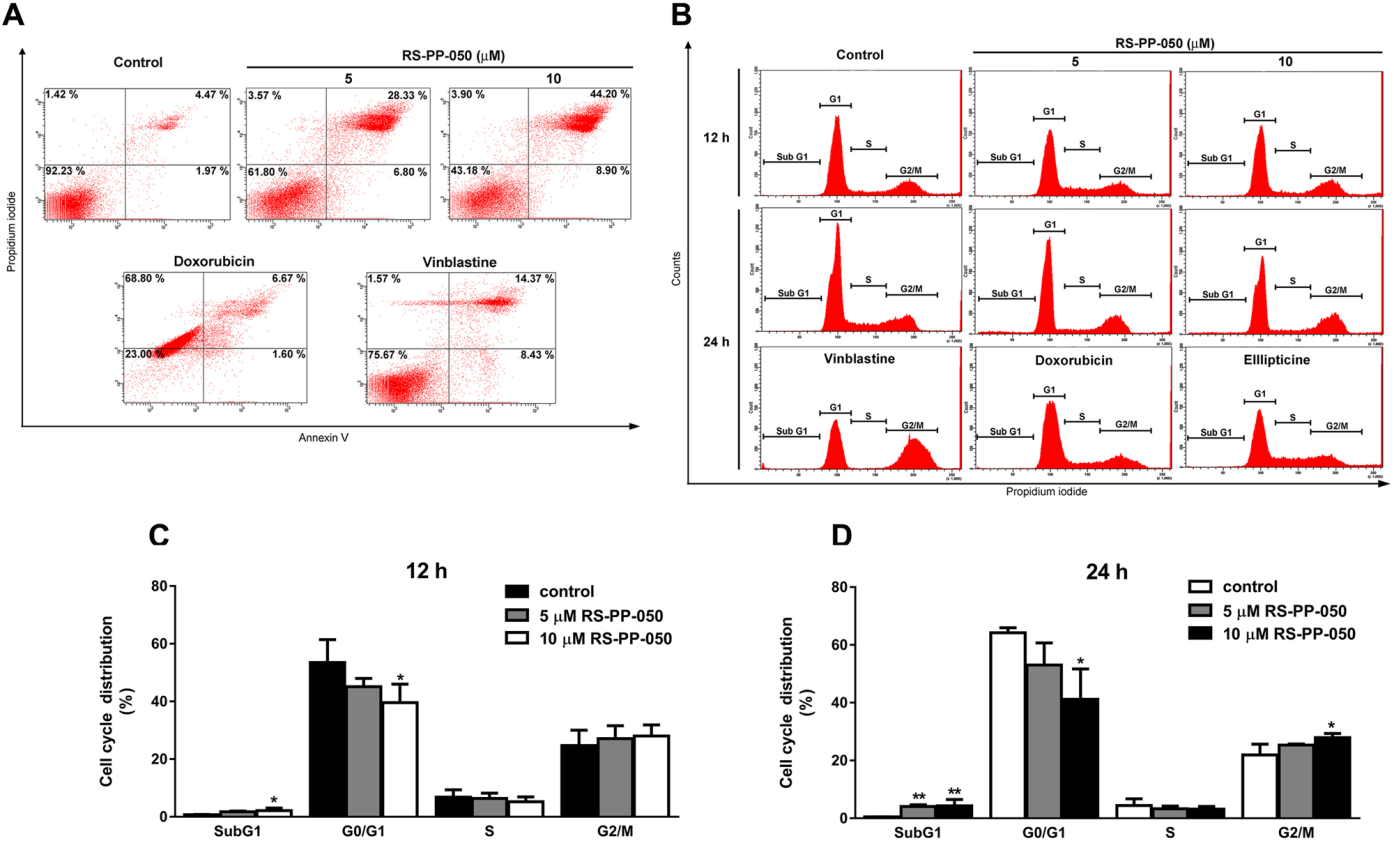
Induction of apoptosis and cell cycle arrest by RS-PP-050. (**A**) Representative FACS analysis showing RS-PP-050 induced apoptosis in HT-29 cells after treatment with RS-PP-050 (5–10 µM) or positive controls; vinblastine (5 µM) and doxorubicin (10 µM) for 24 h. After double-staining with annexin V and PI, cells were subjected to flow cytometry analysis. (**B**) Representative DNA histogram showing cell cycle arrest of HT-29 cells after treatment with the indicated concentrations of RS-PP-050 or positive controls for 12–24 h. (**C**,**D**) Percentages of DNA content in each cell cycle phase are shown in each panel. Data are means ± S.E.M compared with the vehicle control (n = 3) (*P < 0.05, **P < 0.01).

To determine the ability of RS-PP-050 to inhibit cell growth and survival, the effect of RS-PP-050 on the induction of apoptotic cell death and cycle arrest in HT-29 cells were analyzed by flow cytometry. Vinblastine, doxorubicin and ellipticine were used as positive controls. The effect of RS-PP-050 on apoptotic cell death was evaluated by double staining with annexin V-FITC and propidium iodide (PI) to indicate the externalization of phosphatidylserine on the surface of the cell membrane. RS-PP-050 increased the percentage of apoptotic populations at both the early and late phases after 24 h of treatment in a dose-dependent manner (Fig. 2A). Vinblastine and doxorubicin also induced the apoptosis of HT-29 cells in a similar manner. These findings suggest that RS-PP-050 induced HT-29 cell death via apoptosis.

To further investigate the effect on cell cycle distribution, the relative proportions of DNA content at different stages (sub G1, G1, S and G2/M) were determined in HT-29 cells. After 12 h of exposure to 10 μM RS-PP-050, there was a significant reduction in the cell populations in the G1 phase, and a slight increase in the populations in the G2/M phase (Fig. 2B,C). In contrast, after 24 h of exposure, RS-PP-050 significantly reduced the populations in the G1 phase and increased populations in the G2/M phase (G1, from 64.20 ± 1.0% in DMSO control to 41.17 ± 6.1%; G2/M, from 21.93 ± 2.2% in DMSO control to 27.87 ± 0.9%, respectively) (Fig. 2B,D). The increase of cell number at sub G1 phase reflecting cells undergoing apoptosis also displayed both dose and treatment time-dependence (Fig. 2B–D). The positive controls vinblastine, doxorubicin, and ellipticine also caused cell cycle arrested at the G2/M phase after 24 h of treatment. Altogether, these results suggest that RS-PP-050 inhibits HT-29 cell growth and survival through cell cycle arrest and induction of apoptosis.

### Up-regulation of apoptotic proteins and induction of intracellular ROS by RS-PP-050

**Figure 3:**
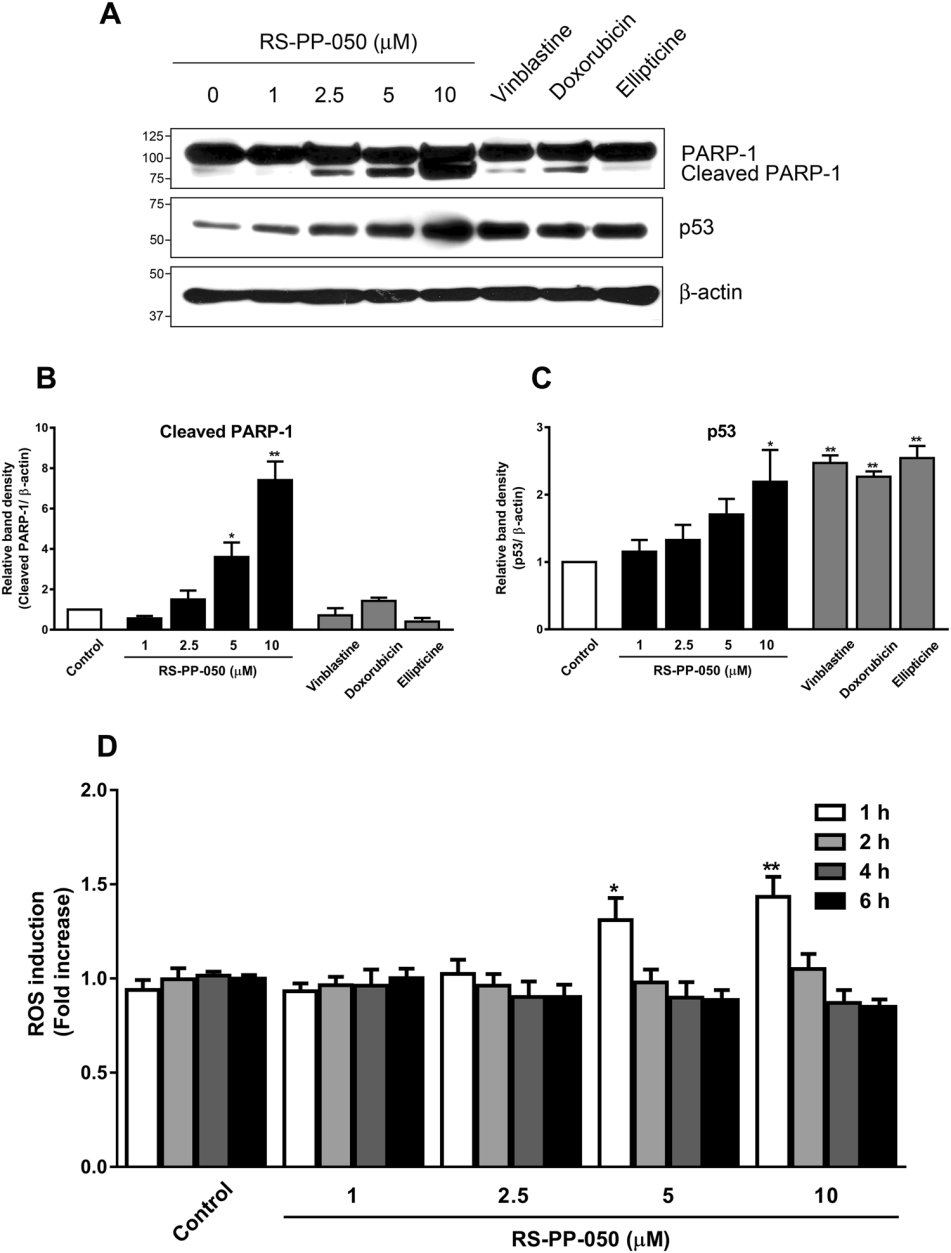
Up-regulation of apoptotic proteins and induction of intracellular ROS by RS-PP-050. (**A**) Immunoblot representing the increase in protein expression of cleaved PARP-1 and p53 after treatment with RS-PP-050 or the positive controls; vinblastine (5 µM), doxorubicin (10 µM), and ellipticine (10 µM) for 24 h. β-actin serves as a loading control. For the cropped blots, protein samples were run under same conditional treatments and processed in parallel. Full-length blots are presented in Supplementary Fig. S1. (**B**,**C**) Bar graphs represent the normalized band intensities of cleaved PARP-1 and p53. Data are means ± S.E.M compared with the vehicle control (n = 3) (*P < 0.05, **P < 0.01). (**D**) The induction of intracellular ROS production in HT-29 cells determined with DCFDA fluorescent probe after treatment with RS-PP-050 at various concentrations and periods. Data are means ± S.E.M compared with the vehicle control (n = 3) (*P < 0.05, **P < 0.01).

A variety of proteins are involved in the processes of apoptotic cell death and cell cycle distribution. In the present study, the expression of PARP-1was determined by western blotting. PARP-1 is an ADP-ribosylating nuclear enzyme responsible for maintaining genome integrity. It is activated upon the induction of DNA strand breaks as well as DNA damage. The expression of cleaved PARP-1 was substantially increased by RS-PP-050 at 5 and 10 μM concentrations (Fig. 3A,B). This induction of PARP-1 expression was greater than that induced by the positive control compounds at the similar concentrations. Moreover, the expression of p53, a tumor suppressor protein involved in regulation of apoptosis under genotoxic stimulation was also determined. Similarly, p53 expression was up-regulated in a dose-dependent manner following treatment with RS-PP-050 (Fig. 3A,C). In accordance with the results of the flow cytometry studies, these findings suggest that the activation of PARP-1 and p53 is a direct result of DNA damage induced by RS-PP-050 in HT-29 cells.

As induction of reactive oxygen species (ROS) production is one of the important characteristics of effective anti-cancer agents, we then determined whether the cytotoxic effects of RS-PP-050 involves the generation of ROS by using 2′,7′-Dichlorofluorescein diacetate (DCFDA), a dye that can be hydrolyzed by intracellular esterases and oxidized by ROS. ROS induction by RS-PP-050 in HT-29 cells was found to be both concentration- and time-dependent. Upon treatment with increasing RS-PP-050 concentrations, ROS production was increased and then decreased to baseline (Fig. 3D). We found that exposure of HT-29 cells to 1 µM RS-PP-050 resulted in a maximal increase of ROS production at 6 h of incubation. Whereas HT-29 cells treated with higher concentrations of RS-PP-050 showed a rapid increase in ROS production at 1 h after incubation. Taken together, our results suggest that RS-PP-050-induced cell death might also be associated with the induction of intracellular ROS generation.

### Inhibition of TCF/LEF reporter activity of Wnt/β-catenin signaling by RS-PP-050

**Figure 4:**
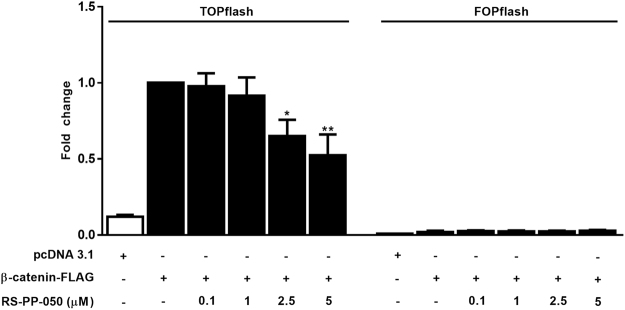
Inhibition of TCF/LEF reporter activity of Wnt/β-catenin signaling by RS-PP-050. The reduction of TCF reporter activity in RS-PP-050-treated cells. HEK293T cells were transiently transfected with β-catenin-FLAG or the empty control pcDNA3.1, and TOPflash or FOPflash, and *Renilla* luciferase reporter plasmids. After transfection, cells were incubated with RS-PP-050 for 24 h. The relative firefly luciferase activity units (RLUs) was then measured and normalized corresponding to *Renilla* luciferase activity. Data are expressed as the fold change compared with β-catenin-transfected cells and represented as means ± S.E.M (n = 3) (*P < 0.05, **P < 0.01).

Aberrant activation of Wnt/β-catenin signaling is a critical step in the initiation and development of CRC. β-catenin is the pivotal mediator of this pathway. To study the effect of RS-PP-050 on the transduction of β-catenin, a luciferase reporter assay was employed. HEK293T cells were transiently transfected with a plasmid for β-catenin expression, and a TOPflash reporter plasmid, a construct containing wild-type TCF/LEF binding sites upstream of either a luciferase gene, or the gene for FOPflash, a negative control reporter to indicate the specificity on inhibition of Wnt signaling. In the control, β-catenin induced transcription of the transfected TOPflash reporter in HEK293T cells approximately 5-fold compared with cells transfected pcDNA3.1. After treatment, RS-PP-050 at concentrations of 2.5, and 5 µM significantly inhibited the TCF/LEF activity in the β-catenin-overexpressed cells (Fig. 4). In contrast, the luciferase activity in cells transfected with FOPflash plasmid, containing a mutation in the TCF/LEF binding site was not affected by the analogue. These results suggest that RS-PP-050 essentially inhibits β-catenin-mediated transcriptional activity of the canonical Wnt signaling pathway.

### Down-regulation of the expression of Wnt downstream target genes by RS-PP-050

**Figure 5:**
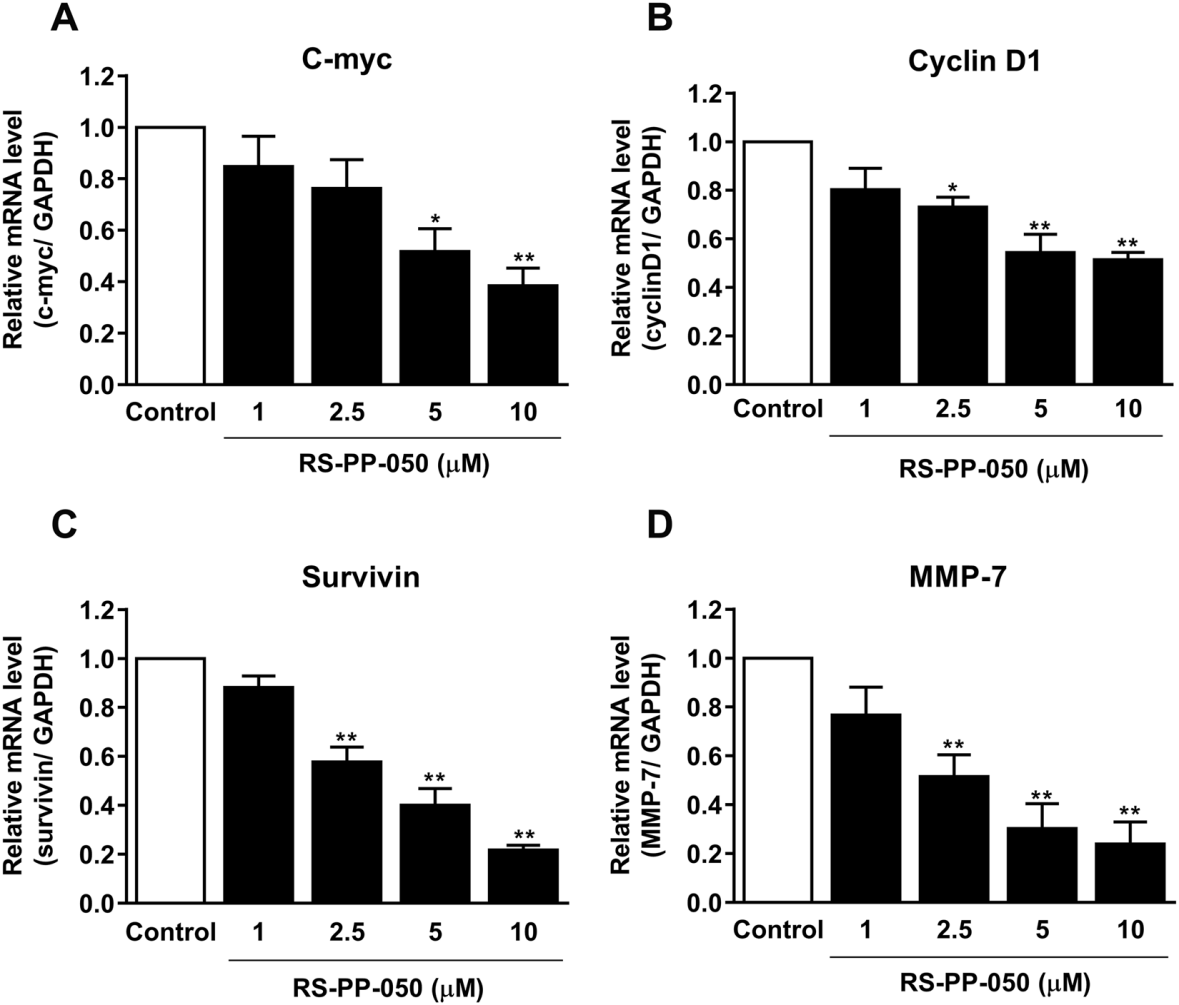
Down-regulation of the expression of Wnt downstream target genes by RS-PP-050. Quantitative real-time PCR showing the concentration-dependent reduction in mRNA expression of Wnt target genes: (**A**) c-myc, (**B**) cyclin D1, (**C**) survivin, and (**D**) MMP-7 in HT-29 cells after treatment with RS-PP-050 for 24 h. The relative mRNA expression was quantified and normalized with GAPDH. Data are means ± S.E.M. compared with the vehicle control (n = 3) (*P < 0.05, **P < 0.01).

We further assessed the suppressive effect of RS-PP-050 on Wnt/β-catenin signaling by determining the expression of endogenous Wnt target genes. By real-time PCR, it was found that RS-PP-050 decreased the mRNA levels of c-myc and cyclin D1 in HT-29 cells in a dose-dependent manner after 24 h of treatment (Fig. 5A,B). In addition, the analogue also decreased two more specific Wnt target genes; survivin and MMP-7 (Fig. 5C,D). The reduction in the mRNA expressions of Wnt target genes was also found in other colon cancer cell lines, SW480 and HCT116 cells (see Supplementary Figs S6 and S7). Along with the reduction of the TOPflash luciferase activity, these data strongly indicate that RS-PP-050 inhibition of β-catenin transcription is mediated through Wnt signaling.

### Inhibition of the expression of Wnt/β-catenin proteins by RS-PP-050

**Figure 6:**
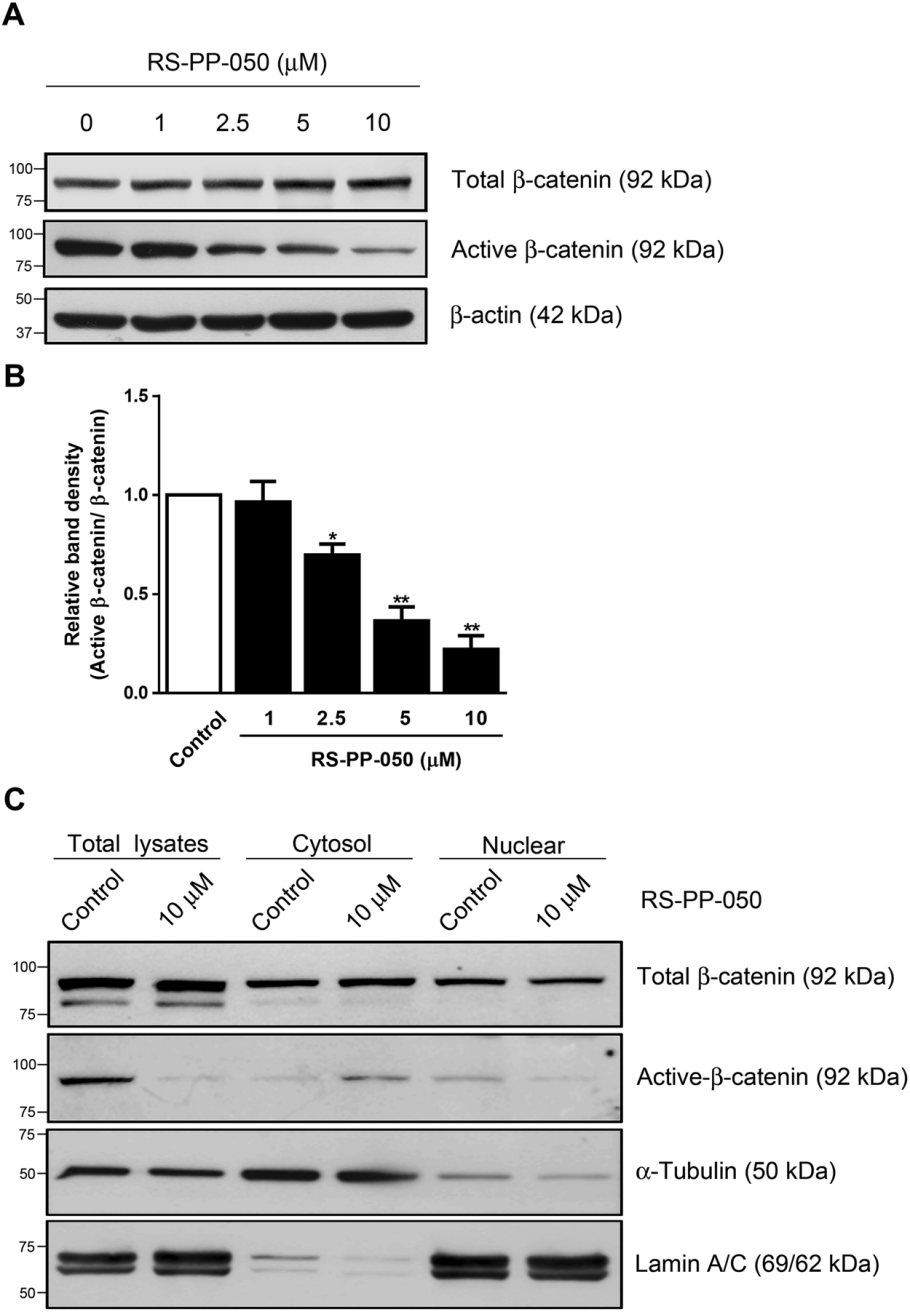
Inhibition of the expression of Wnt/β-catenin proteins by RS-PP-050. (**A**) Immunoblot representing the decrease in protein expression of the active form of β-catenin in HT-29 cells after treatment with RS-PP-050 for 24 h. β-actin serves as a loading control. (**B**) Bar graph represents the normalized band intensities of the active forms of β-catenin to total β-catenin. Data are means ± S.E.M compared with the vehicle control (n = 3) (*P < 0.05, **P < 0.01). (**C**) Immunoblot representing the inhibition in protein expressions of active form of β-catenin in nuclear extracts of HT-29 cells after treatment with RS-PP-050 (10 μM) for 24 h. α-Tubulin, and lamin A/C serve as a cytosolic and a nuclear marker, respectively. For the cropped blots in Fig. 6A,C, protein samples were run under same conditional treatments and processed in parallel. Full-length blots are presented in Supplementary Fig. S2.

To further investigate the inhibitory mechanisms of RS-PP-050 on the regulation of β-catenin transcriptional activity, the changes in expression of Wnt proteins including total and active β-catenin were determined by immunoblotting. Following treatment with RS-PP-050 for 24 h, the expression of total β-catenin protein was not affected, but only the levels of the active form were decreased as compared to the control in HT-29 cells (Fig. 6A,B). These results suggest that RS-PP-050 affects the activation of β-catenin rather than the expression at protein level.

As only the accumulation of a non-phosphorylated β-catenin in the nucleus results in the transactivation of Wnt target genes, we examined whether the inhibitory effect of RS-PP-050 was related to changes in the subcellular localization of β-catenin. The intracellular distribution of β-catenin between the cytoplasm and nucleus was verified by western blotting. RS-PP-050 substantially decreased the levels of active β-catenin present in the nuclear fraction, compared to control, corresponding to the results found in the total lysates (Fig. 6C). While the increased active β-catenin was observed in the cytosolic fraction, which is possibly to account for the reduction of the protein in nucleus. The total cytosolic expression of β-catenin (both phosphorylated and non-phosphorylated) was not significantly altered by the analogue. Taken together, these findings suggest that RS-PP-050 specifically affects the nuclear accumulation of intracellular β-catenin to abrogate the Wnt transcriptional activation.

### Suppression of Wnt/β-catenin signaling through a GSK-3β-independent mechanism by RS-PP-050

**Figure 7:**
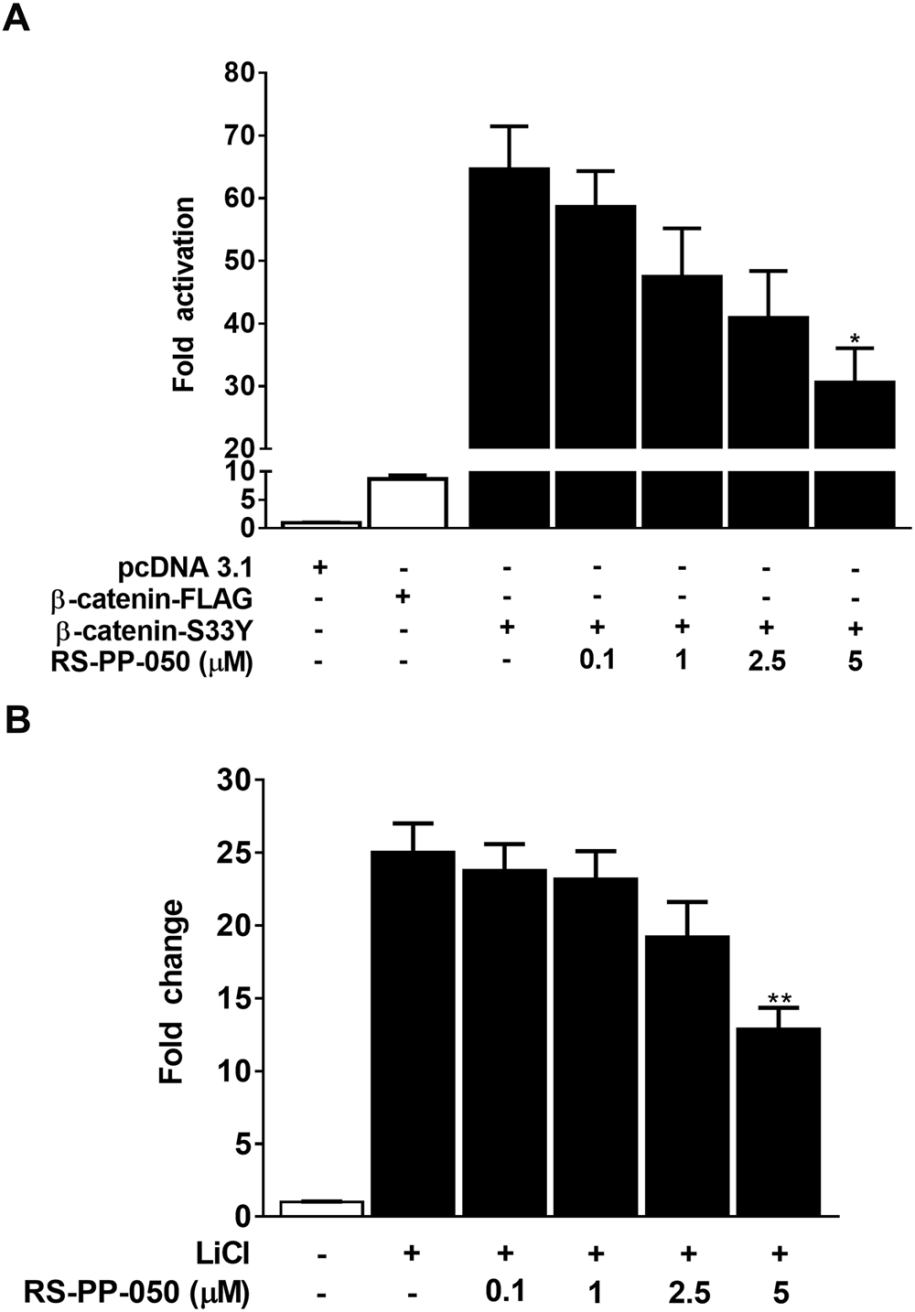
Suppression of Wnt/β-catenin signaling through a GSK-3β-independent mechanism by RS-PP-050. (**A**) HEK293T cells were transiently transfected with a plasmid of wild-type β-catenin (β-catenin-FLAG) or a constitutively active β-catenin mutant (S33Y), TOPflash, and *Renilla* luciferase reporter plasmids. After transfection, cells were incubated with RS-PP-050 for 24 h. The relative firefly luciferase activity units (RLUs) was then measured and normalized corresponding to *Renilla* luciferase activity. Data are expressed as the fold change compared with pcDNA3.1-transfected cells and represented as means ± S.E.M (n = 3) (*P < 0.05). (**B**) HEK293T cells were transiently transfected with TOPflash and *Renilla* luciferase reporter plasmids. After transfection, cells were pretreated with 30 mM LiCl for 16 h, and then co-treated with RS-PP-050 for 24 h. The relative firefly luciferase activity units (RLUs) was then measured and normalized corresponding to *Renilla* luciferase activity. Data are expressed as the fold change compared with LiCl-untreated cells and represented as means ± S.E.M (n = 3) (**P < 0.01).

Because the stability of intracellular β-catenin is tightly regulated by GSK3β, a negative regulator of Wnt signaling, we next investigated the impact of RS-PP-050 on the function of GSK-3β. By using the luciferase reporter assay, HEK293T cells were transiently co-transfected with the same TOPflash reporter plasmid construct containing wild-type TCF/LEF binding sites upstream of a luciferase gene along with and β-catenin (wild-type) or S33Y (mutant β-catenin) plasmids. The luciferase activity in cells transfected with the constitutively active β-catenin mutant (S33Y) was much greater than in the cells transfected with wild-type β-catenin (Fig. 7A). Surprisingly, RS-PP-050 treatment resulted in a dose-dependent decrease in TOPflash luciferase activities in both cells overexpressing wild-type and mutant (S33Y) β-catenin. Treatment of the HEK293T reporter cells with LiCl, a GSK-3 inhibitor, which blocks GSK-3β activity, increases the TCF/LEF luciferase activity, but this effect is inhibited by RS-PP-050 (Fig. 7B). These results provide evidence suggesting that the inhibitory effect of RS-PP-050 on β-catenin activity might be mediated through a GSK-3β-independent mechanism.

Mutations of APC and β-catenin genes in Wnt signaling frequently lead to overactivation of β-catenin transcription^11^. Thus, we also examined whether RS-PP-050 downregulated β-catenin through these genetic alterations. Two CRC cell lines carrying the different mutations were used: SW480 (mutant APC, wild-type β-catenin) and HCT116 (wild-type APC, mutant β-catenin). Treatment with RS-PP-050 reduces protein expression of both total and active forms of β-catenin in SW480 cells and particularly reduces the expression of the active form in HCT116 in a concentration-dependent manner (see Supplementary Fig. S5). These results indicate that inhibition of Wnt signaling by RS-PP-050 is mediated independently of APC or β-catenin.

### Inhibition of the nuclear translocation of phospho-β-catenin (Ser675) by RS-PP-050

**Figure 8:**
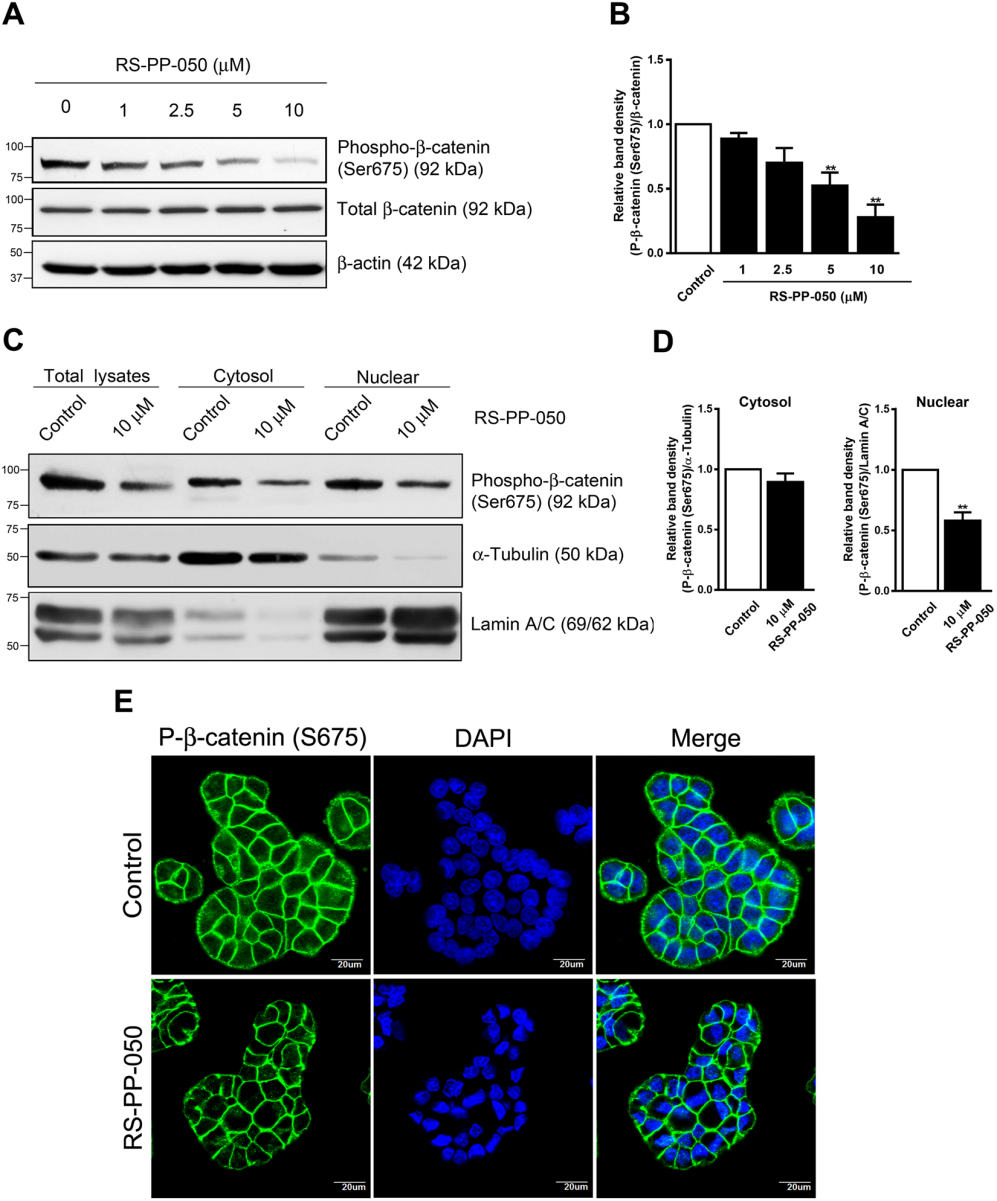
Inhibition of the nuclear translocation of phospho-β-catenin (Ser675) by RS-PP-050. (**A**) Immunoblot representing the decrease in protein expression of phospho-β-catenin (ser675) in HT-29 cells after treatment with RS-PP-050 for 24 h. β-actin serves as a loading control. (**B**) Bar graph represents the normalized band intensities of phospho-β-catenin (ser675) to total β-catenin. Data are means ± S.E.M compared with the vehicle control (n = 4) (**P < 0.01). (**C**) Immunoblot representing the inhibition in protein expression of phospho-β-catenin (ser675) in nuclear extracts of HT-29 cells after treatment with RS-PP-050 (10 μM) for 24 h. α-Tubulin, and lamin A/C serve as a cytosolic and a nuclear marker, respectively. (**D**) Bar graphs represent the normalized band intensities of phospho-β-catenin (ser675) to α-Tubulin, and lamin A/C. Data are means ± S.E.M compared with the vehicle control (n = 3) (**P < 0.01). For the cropped blots in Fig. 8A,C, protein samples were run under same conditional treatments and processed in parallel. Full-length blots are presented in Supplementary Fig. S3. (**E**) Immunofluorescent image demonstrating the reduction of nuclear accumulation of phospho-β-catenin (ser675) (green) in HT-29 cells after treatment with RS-PP-050 (5 μM) for 12 h (Lower panel). DAPI was used as a nuclei marker (blue). The slides were visualized with a confocal laser microscopy. Scale bars = 20 μM.

The accumulation of nuclear β-catenin is a key process in the activation of Wnt signaling and the expression of target genes. Recent findings have reported that the phosphorylation of β-catenin at serine 675 by PKA increases its nuclear translocation independently of GSK-3β activation^13,14^. We therefore investigated whether the phosphorylation of β-catenin is also impacted by RS-PP-050-mediated β-catenin expression and nuclear localization. Interestingly, the expression of phosphorylated β-catenin at serine 675 residue was downregulated by RS-PP-050 in HT-29 cells (Fig. 8A,B). This result was confirmed by collecting both cytosolic and nuclear fractions from treated cells and subjecting them to western blot analysis. Surprisingly, the expression of the phosphorylated β-catenin is dramatically reduced in the nuclear extract after treatment with RS-PP-050 (Fig. 8C,D). To further verify that RS-PP-050 decreases Wnt/β-catenin activity by interfering with the nuclear translocation of β-catenin, its localization was studied by immunofluorescence. Phosphorylated β-catenin (ser675) was accumulated in the membrane, cytoplasm, and nucleus of untreated cells (Fig. 8E). Consistent with the western blot results, we clearly demonstrate that treatment with RS-PP-050 extensively reduced the translocation of β-catenin from the plasma membrane and cytoplasm to the nucleus. These data suggest that RS-PP-050 potently inhibits β-catenin phosphorylation on the C-terminus at ser675, resulting in reduced nuclear translocation of the phospho-β-catenin, thereby attenuating Wnt/β-catenin activity.

## Discussion

Here, we demonstrated that our andrographolide analogue, RS-PP-050 effectively inhibited TCF/LEF transcriptional activity of canonical Wnt/β-catenin signaling. Accordingly, the analogue substantially suppresses the expression of Wnt-responsive genes including c-myc, cyclin D1, survivin, and MMP-7. However, the inhibitory effect of RS-PP-050 on the Wnt/β-catenin inactivation was independent of phosphorylation by GSK-3β, a negative regulator of Wnt. RS-PP-050 inhibited the phosphorylation of β-catenin at ser675 which is critical for nuclear translocation. Indeed, the expression and localization of phosphorylated β-catenin in the nucleus were decreased in treated cells. These findings reveal the underlying mechanism of the anti-cancer activity of RS-PP-050, demonstrating that its activity is mediated through interfering with the phosphorylation of β-catenin (ser675), which subsequently abrogates the transcriptional activity of Wnt/β-catenin signaling inside the nucleus. This is the first evidence showing that the anti-cancer activity of an andrographolide analogue is on the level downstream of GSK-3β toward the nucleus of Wnt signaling pathway.

In the present study, RS-PP-050 suppressed the TCF/LEF transcriptional activity of Wnt signaling in wild-type β-catenin-overexpression HEK293T reporter cells (Fig. 4). As a result, it reduced the expression of Wnt downstream target genes including c-myc, cyclin D1, survivin, and MMP-7, which play important roles in facilitating cell cycle distribution, cell growth, survival and progression. The underlying mechanism of the reduced Wnt activity was further explored. In the Wnt-off state of a canonical Wnt/β-catenin signaling, GSK-3β is a negative regulator inhibiting β-catenin activity by phosphorylating the N-terminal of β-catenin at ser33/ser37/thr41 leading to its degradation in the proteasome. Surprisingly, RS-PP-050 did not affect the phosphorylation-mediated activity to degrade β-catenin of GSK-3β as it causes a decrease in the luciferase activity in cells transiently transfected with S33Y, a mutant β-catenin which is resistant to GSK-3β-mediated phosphorylation (Fig. 7A). Moreover, by using a GSK-3β inhibitor (LiCl), β-catenin activity was consistently reduced by RS-PP-050 (Fig. 7B). All of the data point to a GSK-3β-independent mechanism of RS-PP-050 activity.

The stabilization of β-catenin is controlled by important components of a destruction complex including APC and CK1^28^. Genetic alterations of APC account for 85% of sporadic CRC cases^29^. While alterations of a tumor suppressor CK1, which phosphorylates the N-terminal of β-catenin at ser45 to prime GSK-3β function for subsequent degradation of β-catenin, is linked to the development of colon tumors^30^. By using CRC cell lines, SW480, and HCT116 cells which harbor a mutated form of APC, and CK1, respectively, RS-PP-050 was able to reduce β-catenin stabilization, indicating that RS-PP-050-mediated β-catenin degradation is not associated with APC or CK1. Thus, inhibition of downstream Wnt/β-catenin signaling is more likely to be a potential target for RS-PP-050 action.

Different phosphorylation sites on β-catenin have different effects on β-catenin activities^31^. The phosphorylation of β-catenin at ser675 by cAMP/PKA signaling has been reported to be associated with the nuclear translocation of β-catenin^13,14^. This phosphorylation causes β-catenin to interact with its coactivator, CREB-binding protein (CBP) and promotes β-catenin transcriptional activity. In this current study, the inhibition of Wnt signaling by RS-PP-050 is, at least in part, attributed to its targeting of the nuclear translocation of β-catenin by ser675 phosphorylation (Fig. 8). The reduction of Wnt pathway activity by RS-PP-050 was dependent upon the specific phosphorylation of β-catenin at the ser675 residue and did not depend upon the phosphorylation-mediated degradation at residues ser33/ser37/thr41 by GSK-3β in this study.

In addition to interfering with the Wnt signaling pathway which controls cell proliferation, RS-PP-50 also induced apoptotic cell death (Fig. 2A) and up-regulated the expression of apoptotic proteins, PARP-1 and p53 (Fig. 3A–C). Moreover, it decreased the proportion of cells in the G_1_ phase and arrested at cells at the G_2_/M phase (Fig. 2B–D). These findings indicated that RS-PP-050 might cause deleterious damage to the cells through the induction of apoptosis and cell cycle arrest in which loss of DNA integrity cannot be repaired. These events may be associated with oxidative stress as intracellular ROS production was increased in HT-29 cells after treatment (Fig. 3D).

Structurally, the cytotoxicity of andrographolide has been reported to be related to its electrophilic, α-β unsaturated γ-lactone unit which is capable of acting as a Michael acceptor to directly interact with nucleophilic biomolecules inside the cell such as GSH, DNA and proteins^32^. A number of andrographolide analogues have been semi-synthesized to improve their efficacy and potency. RS-PP-050 contains a modification of the side chain of andrographolide with a triphenylmethyl group at C-19. This modification showed a great improvement in the anti-cancer activity as compared with the parent compound^26^. In addition, RS-PP-050 exhibited lower cytotoxic effect against normal human liver cells indicating the selective property of this compound. The reason for the augmentation in the anti-cancer activity of the compound after the addition of the triphenylmethyl group of RS-PP-050 is not yet known. The modification may promote the electrophilic properties of the α, β-unsaturated γ-lactone moiety of andrographolide. However, the substitution of the trityl group of RS-PP-050 increased the LogP value by 6.24-fold over the parent compound (LogP was calculated from http://www.molinspiration.com). Thus, the enhancement in lipophilicity potentially increases the penetration ability of the compound into the cells to efficiently kill cancer cells. This concept has been successfully demonstrated in several anti-cancer compounds modified from andrographolide^33,34^.

In conclusion, an andrographolide analogue RS-PP-050 acts as a potent anti-cancer compound against CRC cell growth by induction of cell cycle arrest and cellular apoptosis. It targets the Wnt signaling pathway in the nucleus by inhibiting β-catenin transcriptional activity and the nuclear accumulation of active β-catenin protein independently of GSK-3β phosphorylation. RS-PP-050 impairs the nuclear translocation of β-catenin with ser675 phosphorylation to inactivate Wnt activity. RS-PP-050 might be a promising chemotherapeutic agent used for targeting Wnt/β-catenin-overexpressing cells in human cancers and other diseases. These findings provide an instructive basis for exploiting a newly identified chemotherapeutic agent in Wnt/β-catenin-overexpressing CRC cells.

## Materials and Methods

### Cell Culture

Human embryonic kidney (HEK293T), Chang liver (human normal liver), and three CRC cell lines, HT-29, HCT116 and SW480 were obtained from the American Type Culture Collection (ATCC). HEK293T and Chang liver cells were maintained in Minimum Essential Media (MEM). HT-29 cells were maintained in Dulbecco’s modified Eagle’s medium: Nutrient Mix F-12 (DMEM/F12). HCT116 and SW480 cells were maintained in Dulbecco’s modified Eagle’s medium (DMEM) (Invitrogen, Carlsbad, CA, USA). All growth media was supplemented with 10% fetal bovine serum (FBS) (Hyclone, Perbio, UT, USA), 100 μg/ml penicillin, and 100 μg/ml streptomycin (Invitrogen, Carlsbad, CA, USA). All cells were cultured at 37 °C in a humidified incubator with 5% CO_2_.

### Compounds and reagents

Andrographolide and a semi-synthetic andrographolide analogue, RS-PP-050 (19-*O*-triphenylmethyl andrographolide) are provided by Asst. Professor Dr. Rungnapha Saeeng (Department of Chemistry, Faculty of Science, Burapha University, Thailand) as previously described as compound no.18^26^. The chemical structures of the compounds can be seen in Supplementary Fig. S4. The following reagents were used: lipofectamine 2000, Alexa Fluor 488 and TRIzol reagent (Invitrogen, Carlsbad, CA,USA); Doxorubicin (Guanyu Bio-tech, Xi’an, Republic of China); Cell proliferation ELISA, BrdU (colorimetric) and DAPI (Roche Diagnostic, Mannheim, Germany); 3-(4,5-dimethylthiazol-2-yl)−2,5-diphenyltetrazolium bromide (MTT), vinblastine, ellipticine and lithium chloride (Sigma-Aldrich, St. Louis, MO); Annexin V-FITC apoptosis detection kit, and propidium iodide (PI)/RNase Staining Buffer (BD Biosciences, SanJose, CA, USA); Dual-luciferase reporter assay (Promega, Madison, WI, USA); iScript^TM^ select cDNA synthesis kit (Bio-Rad, Hercules, CA, USA); SYBR kit (Applied Biosystem, Carlsbad, CA, USA); BCA protein assay kit, protease inhibitor cocktail, RIPA buffer, Super Signal West Pico Chemiluminescent Substrate (Thermo Scientific, Cramlington, UK), and phosphatase inhibitor cocktail (Millipore, Darmstadt, Germany); The following antibodies were used: anti-β-catenin (clone H-102) and anti-lamin A/C (Santa Cruz Biotechnology, CA, USA); anti-active-β-catenin (clone 8E7) (Millipore, Darmstadt, Germany); anti-Phospho-β-catenin (Ser675), anti-Phospho-GSK-3β (Ser9) and anti-GSK-3β (Cell Signaling Technology, Danvers, MA, USA); anti-β-actin and anti-α-tubulin (Sigma). The TCF/ LEF reporter plasmid (TOPflash) and the negative control reporter plasmid (FOPflash), wild-type β-catenin-FLAG and mutant β-catenin S33Y plasmids were described previously^35^.

### Cell Viability Assay

Cells were plated at a density of 1 × 10^4^ cells/well in 96-well plates and treated with various concentrations of the test compounds for 24, 48 and 72 h. At the indicated time points, an MTT assay was performed to assess viability. The medium was removed and substituted with MTT working solution and incubated for 4 h at 37 °C in 5% CO_2_ in humidified atmosphere. The medium was then replaced with DMSO and the optical density was measured at a wavelength of 540 nm using a Multiskan™ GO Microplate Spectrophotometer (Thermo Scientific, Cramlington, UK).

### Cell proliferation assay

Cells were plated at a density of 1 × 10^4^ cells/well into 96-well plates for 24 h, and treated with various concentrations of RS-PP-050 for 24 h. After incubation, BrdU labeling solution was added, and the cells were fixed and incubated with an anti-BrdU peroxidase conjugated antibody using the cell proliferation ELISA BrdU kit according to the manufacturer’s instructions. The reaction was stopped with 1 M H_2_SO_4_ and the absorbance was measured at a wavelength of 450 nm (reference wavelength: 690 nm) using a Multiskan™ GO Microplate Spectrophotometer (Thermo Scientific, Cramlington, UK).

### Clonogenic assay

Cells treated with RS-PP-050 at various concentrations were replated in 6-well plates at a density of 1 × 10^3^ cells/well in the growth medium. Plates were further incubated for 15 days with changing of medium every 2–3 days until colonies were large enough to be visualized. Colonies were fixed with methanol and stained with 0.5% crystal violet for 1 h and dried at room temperature. Pictures of cells were taken and the colonies were counted using Image J software (NIH, Bethesda, MA, USA).

### Flow cytometry Analysis

To study cellular apoptosis, cells were seeded in 10-cm dishes at a density of 2 × 10^6^ cells/dish and treated with different compounds for 24 h. Later, cells were harvested by trypsinization, centrifuged and washed twice with PBS. The cell pellets were re-suspended in FITC-labeled annexin V and PI for 15 min in dark place. For cell cycle experiments, cells were seeded in 10-cm dishes and treated with different compounds for 12–24 h. Later, cells were collected and fixed with cold 70% ethanol for at least 24 h followed by washing twice with PBS and staining with PI/RNAse solution for 15 minutes in the dark. The stained cells were analyzed using a BD FACS Calibur™ flow cytometer. The different stages of cell death and the percentage of DNA content in each phase were quantified using BD FACS Diva software version 6.1.1 (BD Bioscience).

### ROS measurement

Intracellular ROS levels were measured using 2′,7′-Dichlorofluorescein diacetate (DCFDA). Cells were plated at a density of 1 × 10^4^ cells/well into 96-well plates for 24 h. Cells were then treated with various concentrations of RS-PP-050 at the indicated time points in a 96-well plate. The medium was removed and 20 μM of DCFDA in DPBS were added to each well for 30 min at 37 °C in the dark. Following the incubation, cells were washed three times with DPBS and immediately analyzed at 485 and 535 nm using TECAN Spark 10 M microplate reader (Männedorf, Switzerland).

### Luciferase reporter assay and Transfection

HEK293T cells were seeded into 96-well plates at a density of 1 × 10^4^ cells/well for 18 h. The cells were then transiently co-transfected with 0.1 μg pcDNA3.1 or β-catenin-FLAG or S33Y plasmid, 0.05 μg TOPflash or FOPflash reporter plasmid and 0.05 μg *Renilla* luciferase reporter plasmid for 24 h by using Lipofectamine 2000 reagent according to the manufacturer’s instructions. RS-PP-050 at various concentrations or 0.1% DMSO vehicle control was added. After 24 h, the cells were lysed and subjected for measurement of firefly luciferase activity using the Dual Luciferase Assay kit. The firefly luciferase activity in each treatment was normalized with *Renilla* luciferase activity as an internal control.

### RNA Extraction and Real-time PCR

Total RNA was isolated using the TRIzol reagent following the manufacturer’s instructions. The purity of the RNA was determined using a NanoDrop 2000C (Thermo Scientific). cDNAs were synthesized by using an iScript™ cDNA synthesis kit according to the manufacturer’s protocol and stored at −20 °C until used. Real time PCR of c-myc, cyclin D1, survivin, and MMP-7 was performed on equal amount of cDNA following the protocol provided with SYBR Green and analyzed with ABI PRISM7500 Sequence Detection System and analytical software (Applied Biosystems, Carlsbad, CA, USA). A panel of PCR primers were designed using NCBI/Primer-Blast follows: CyclinD1, FW 5′-GATCAAGTGTGACCCGGACTG-3′ and RW 5′-CCTTGGGGTCCATGTTCTGC-3′; C-myc, FW 5′-GCAGCGACTCTGAGGAGGAA-3′ and RW 5′ GGCCTCCAGCAGGCAGCACA-3′; Survivin, FW 5′-TGAGAACGAGCCAGACTTGG-3′ and RW 5′ TGTTCCTCTATGGGGTCGTCA-3′; MMP-7, FW 5′-TGGGAACAGGCTCAGGACTA-3′ and RW 5′-TGCATCTCCTTGAGTTTGGCT-3′; GAPDH, FW 5′-ATGCCCCCATGTTCGTCATG-3′ and RW 5′-GCAGGAGGCATTGCTGAT-GA-3′. Samples were analyzed in triplicate and the expression ratio was normalized with GAPDH.

### Western Blotting

The treated cells were harvested and lysed by using RIPA buffer supplemented with protease inhibitors and phosphatase inhibitor cocktail. Cytosolic and nuclear fractions were extracted by using a NE-PER Nuclear and Cytosolic Extraction kit (Thermo Scientific). Proteins were separated by 10% sodium dodecyl sulfate–polyacrylamide gel electrophoresis (SDS-PAGE) and transferred onto PVDF membranes. Blots were blocked with 5% nonfat dry milk and probed overnight at 4 °C with primary antibody. The membranes were then incubated with specific secondary antibodies. The detection of the bands was developed using ECL reagent and imaged with Amersham Hyperfilm^TM^ (GE Healthcare, Buckinghamshire, UK). The densitometry analysis was performed by using ImageJ software (NIH, MA, USA).

### Immunofluorescence microscopy

Cells were seeded on glass cover slips in a separate 24-well plate. After treatment, cells were fixed in methanol at −20 °C, washed with cold PBS containing Ca^2+^/Mg^2+^ (PBS^++^), permeabilized in 0.2% Triton X-100 and blocked with 10% BSA. The slides were incubated for 2 h at room temperature with primary rabbit anti-phospho-Ser675-β-catenin (1:200), followed by a 1 h incubation in the dark with an anti-rabbit Alexa Flour 488 conjugated antibody (1:500) and DAPI for nuclei visualization. Stained cells were imaged with a confocal laser scanning microscope (FV10i, Olympus, Japan) at a magnification of 60x. The images were analyzed using the provided analysis software (FV10-ASW 3.0 Viewer, Olympus, Japan).

### Statistical analysis

All statistical analysis was performed using GraphPad Prism 5.01 (GraphPad Software, San Diego, CA). Data in each group were compared using a two-tailed unpaired student’s t test or one-way ANOVA with Tukey’s post-test. P-values < 0.05 and <0.01 were considered statistically significant.

## Electronic supplementary material

Supplementary Information
