# Supplementary material for: The anti-cancer activity of an andrographolide analogue functions through a GSK-3β-independent Wnt/β-catenin signaling pathway in colorectal cancer cells
Source: Sci Rep. 2018 May 21;8:7924. doi: 10.1038/s41598-018-26278-8 (PMC5962551; doi:10.1038/s41598-018-26278-8)

Supplementary information:

The anti-cancer activity of an andrographolide analogue functions through a GSK-3β-independent Wnt/β-catenin signaling pathway in colorectal cancer cells

Somrudee Reabroi[1], Rungnapha Saeeng[2], Nittaya Boonmuen[1], Teerapich Kasemsuk[2], Witchuda Saengsawang[1], Kanoknetr Suksen[1], Weiming Zhu[3], Pawinee Piyachaturawat[1] & Arthit Chairoungdua[1, 4,*]

[1]Department of Physiology, Faculty of Science, Mahidol University, Bangkok 10400, Thailand.
[2]Department of Chemistry and Center for Innovation in Chemistry, Faculty of Science, Burapha University, Chonburi 20131, Thailand.
[3]Key Laboratory of Marine Drugs, Ministry of Education of China, School of Medicine and Pharmacy, Ocean University of China, Qingdao 266003, China.
[4]Excellent Center for Drug Discovery (ECDD), Mahidol University, Bangkok 10400, Thailand.

[*]Correspondence and requests for materials should be addressed to A.C.
Arthit Chairoungdua, Ph.D
Department of Physiology,
Faculty of Science, Mahidol University,
Rama 6 Road, Bangkok, Thailand 10400.
E-mail: arthit.chi@mahidol.ac.th
Phone: (+662) 2015619
Fax: (+662) 3547154

Supplementary Information:

Supplementary Figures:

Supplementary Figure S1. Full-length blots representing the protein expressions of cleaved PARP-1, p53, and β-actin as shown in Figure 3A.

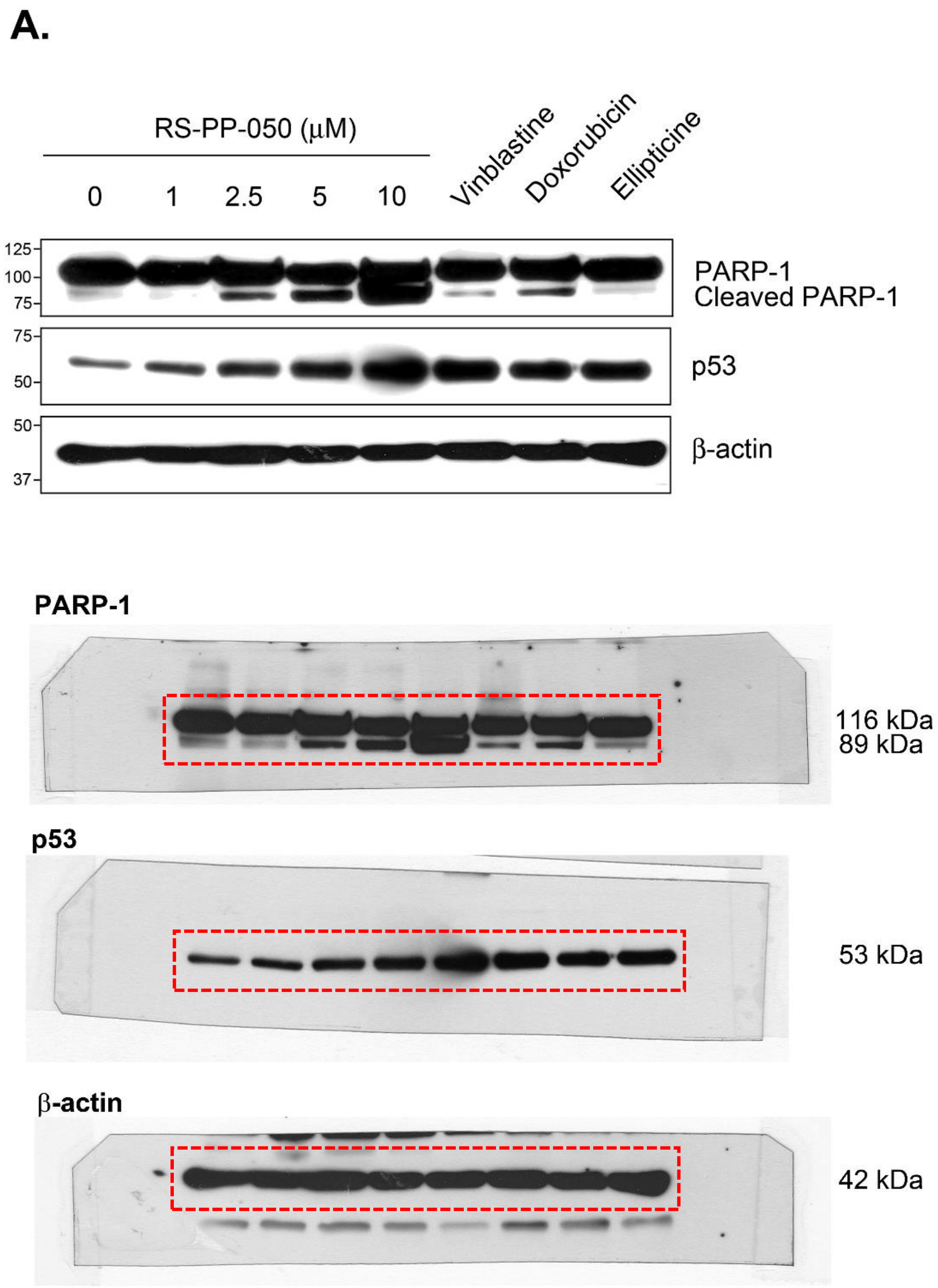

Supplementary Figure S2. Full-length blots representing the protein expressions of total and the active forms of β-catenin, β-actin, α-Tubulin, and lamin A/C as shown in Figure 6A and 6C, respectively.

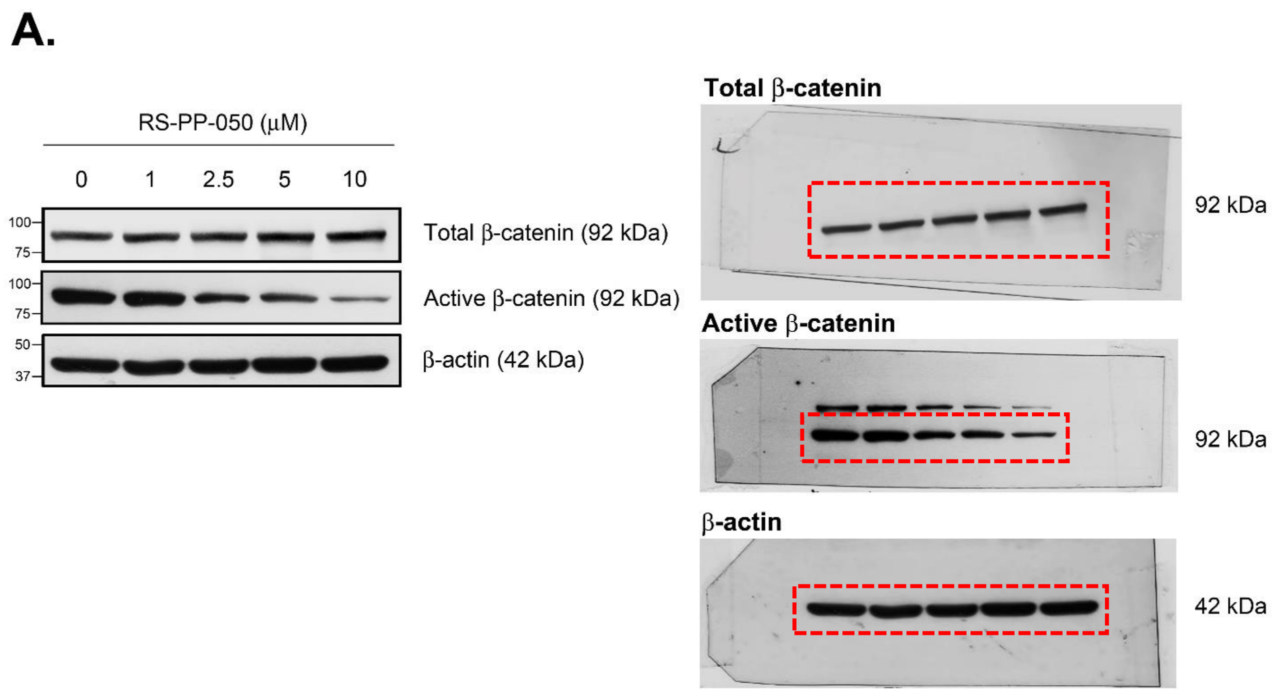

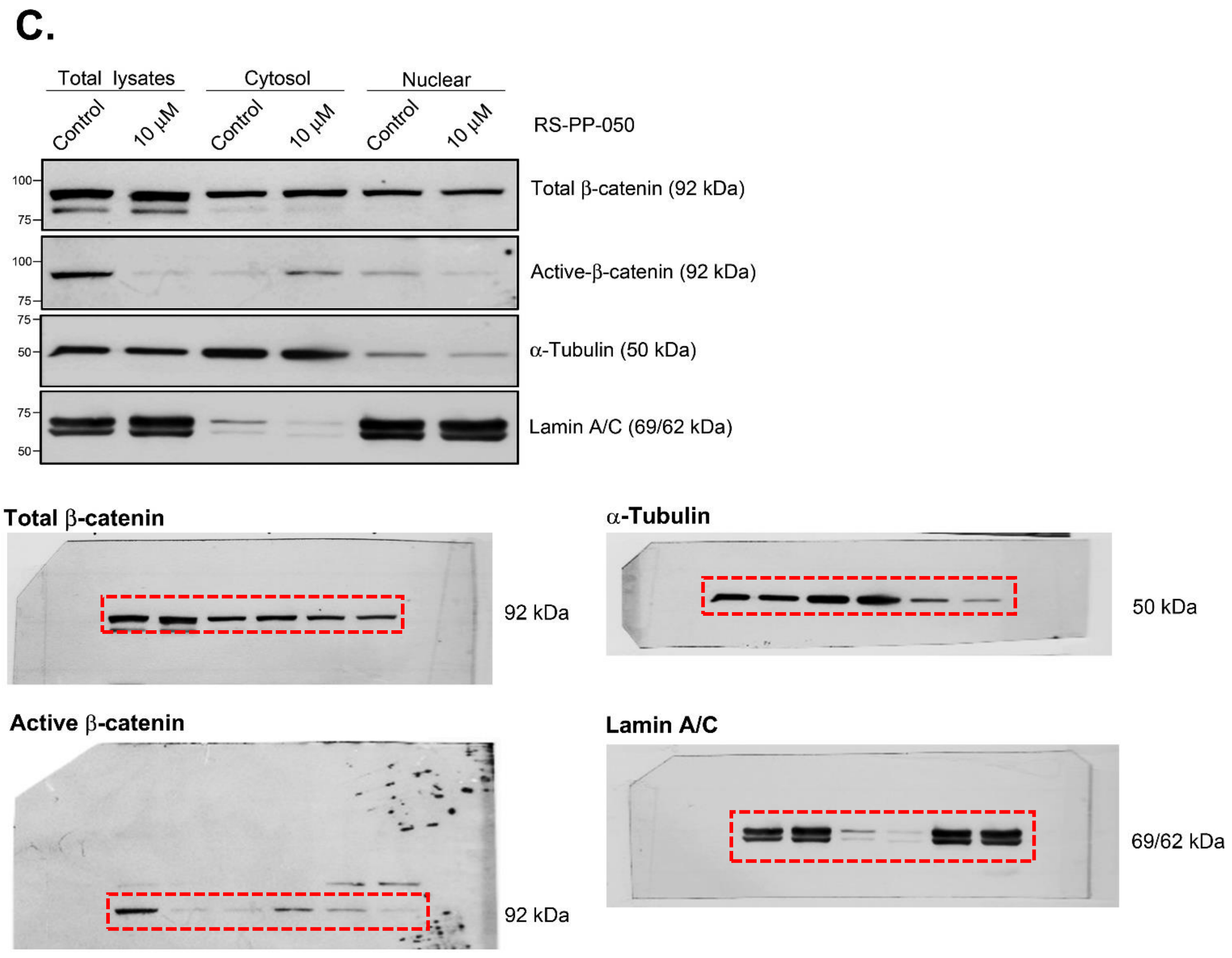

Supplementary Figure S3. Full-length blots representing the protein expression of phospho-β-catenin (ser675), total β-catenin, β-actin, α-Tubulin, and lamin A/C as shown in Figure 8A and 8C, respectively.

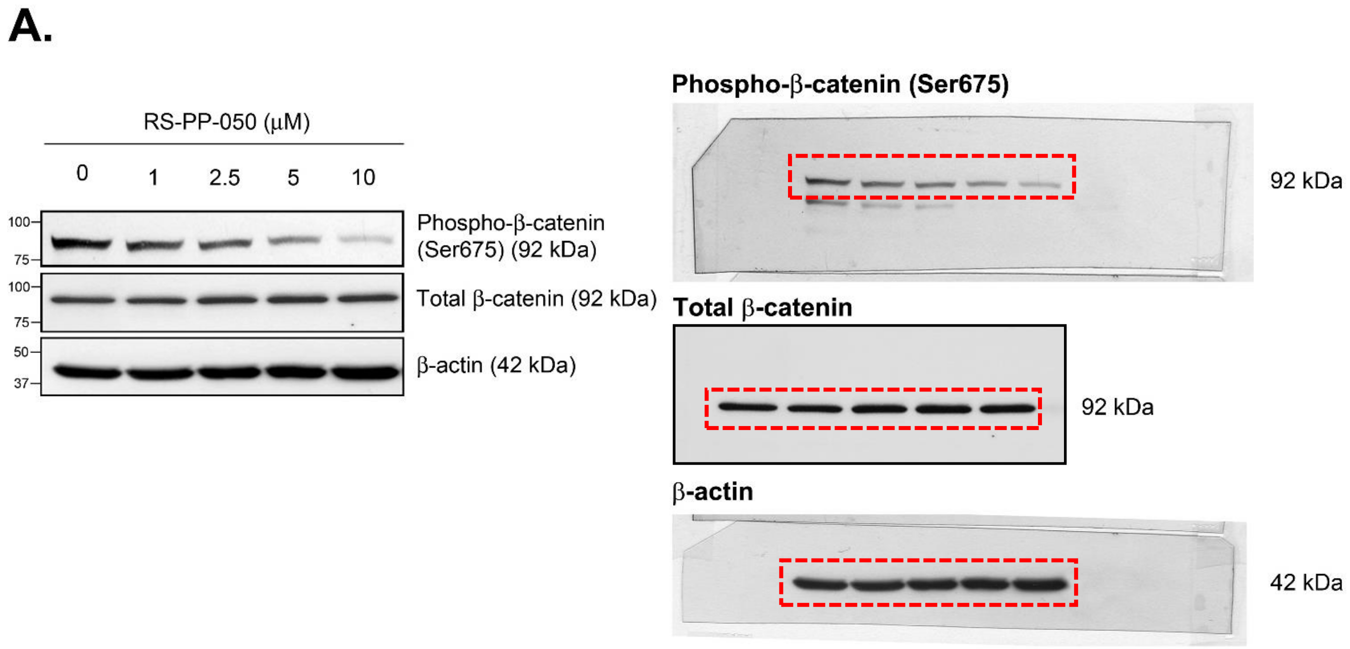

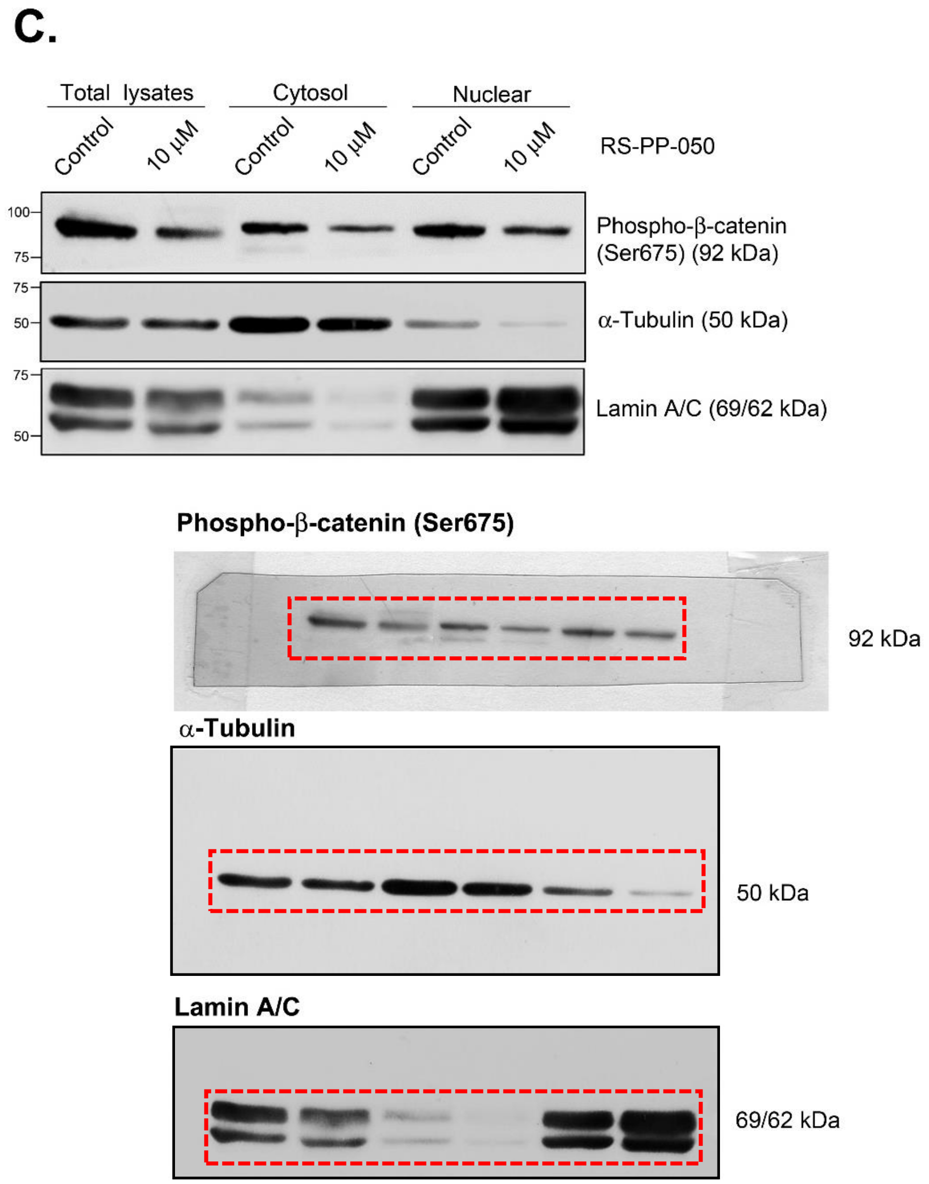

Supplementary Figure S4. Chemical structures of (A) andrographolide, and (B) 19-triphenylmethyl andrographolide (RS-PP-050).

A. Andrographolide

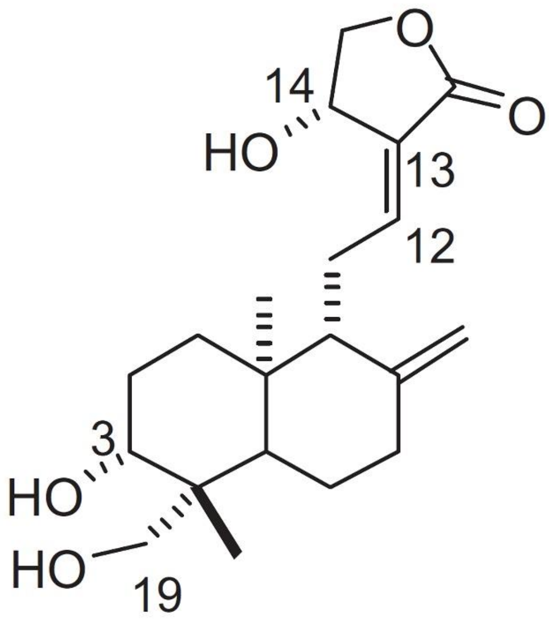

B. 19-*O*-triphenylmethyl andrographolide

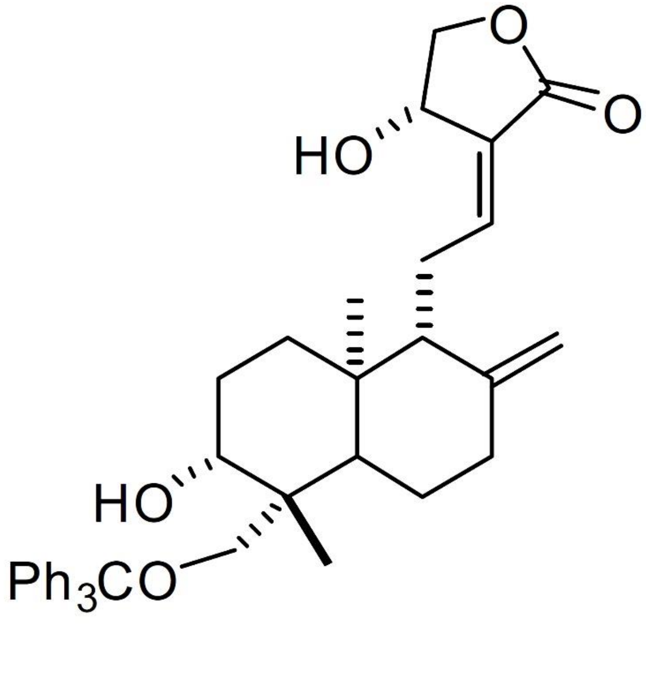

Supplementary Figure S5. The alteration of Wnt protein expression by RS-PP-050 in two CRC cell lines, SW480 and HCT116, is independent of APC or β-catenin. Immunoblot representing the decrease in protein expression of active β-catenin in (A) SW480 and (B) HCT116 cells after treatment with different concentrations of RS-PP-050 for 24 h. β-actin serves as a loading control. For the cropped blots, protein samples were run under same conditional treatments and processed in parallel. Full-length blots are presented in the right panel.

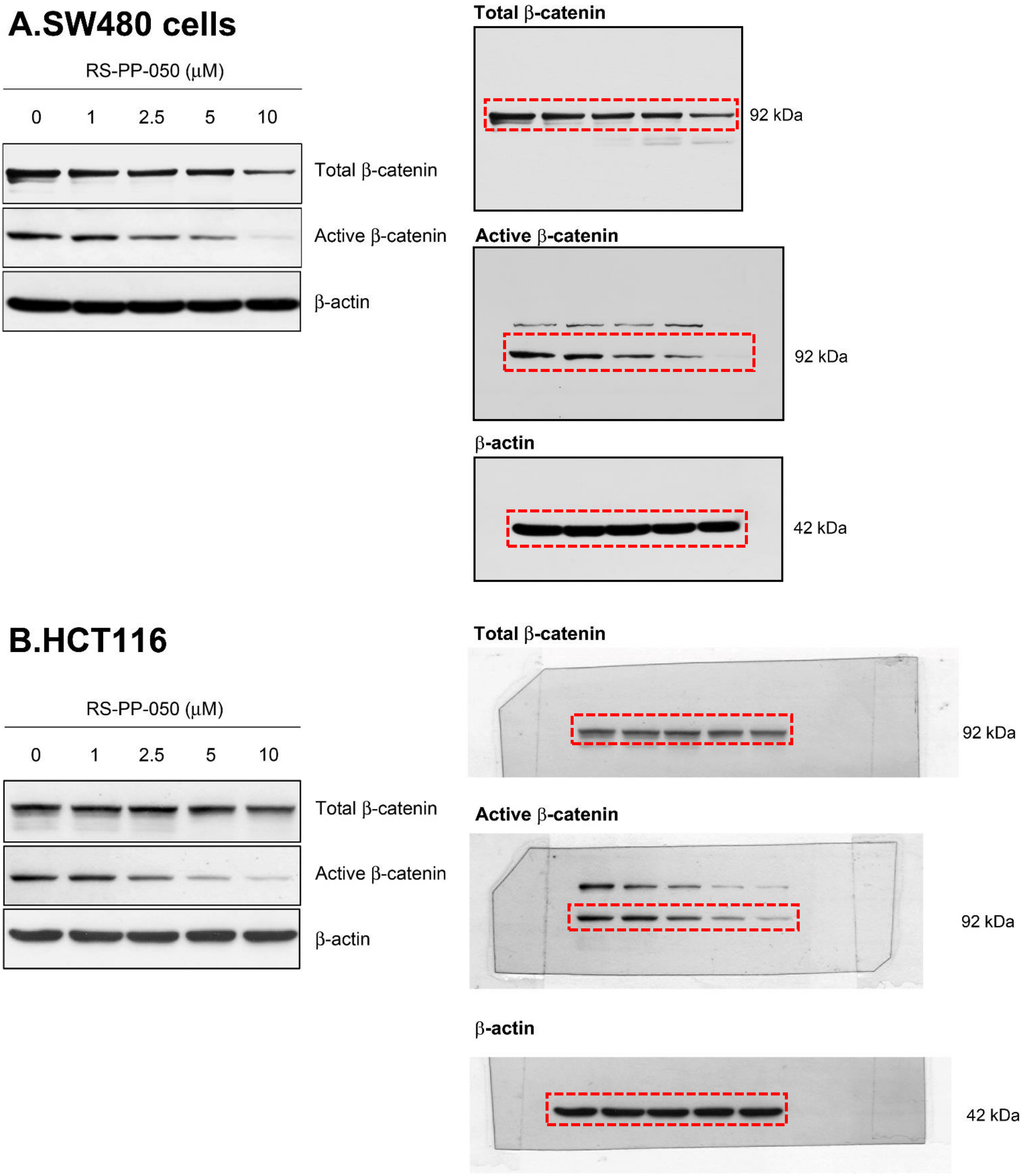

Supplementary Figure S6. Quantitative real-time PCR showing the concentration-dependent reduction in mRNA expression of Wnt target genes: (A) c-myc, (B) cyclin D1, (C) survivin, and (D) MMP-7 in SW480 cells after treatment with RS-PP-050 for 24 h. The relative mRNA expression was quantified and normalized with GAPDH. Data are mean ± S.E.M compared with the vehicle control (n=3) (**P<0.01).

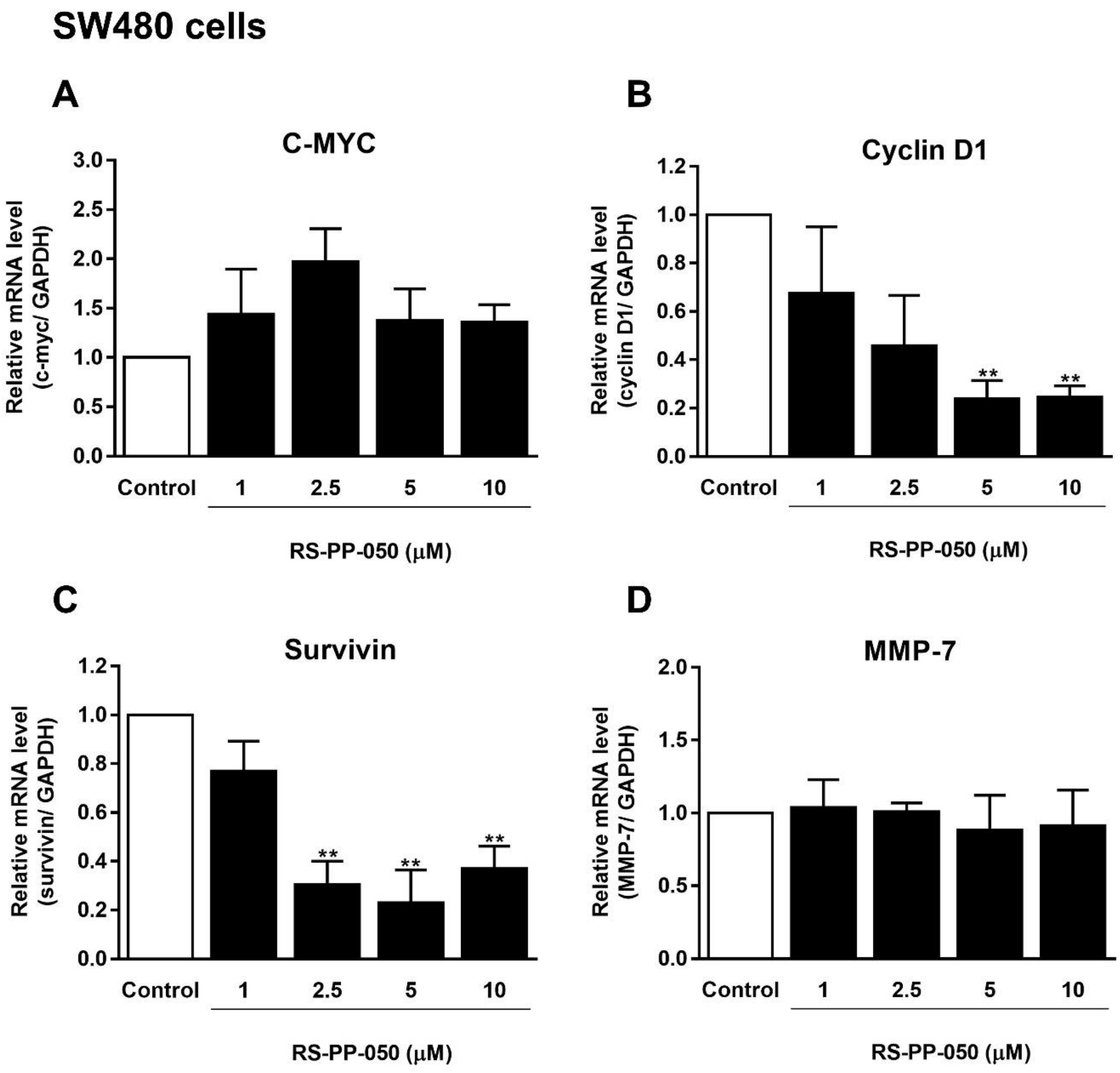

Supplementary Figure S7. Quantitative real-time PCR showing the concentration-dependent reduction in mRNA expression of Wnt target genes: (A) c-myc, (B) cyclin D1, (C) survivin, and (D) MMP-7 in HCT116 cells after treatment with RS-PP-050 for 24 h. The relative mRNA expression was quantified and normalized with GAPDH. Data are mean ± S.E.M compared with the vehicle control (n=3) (*P<0.05, **P<0.01).

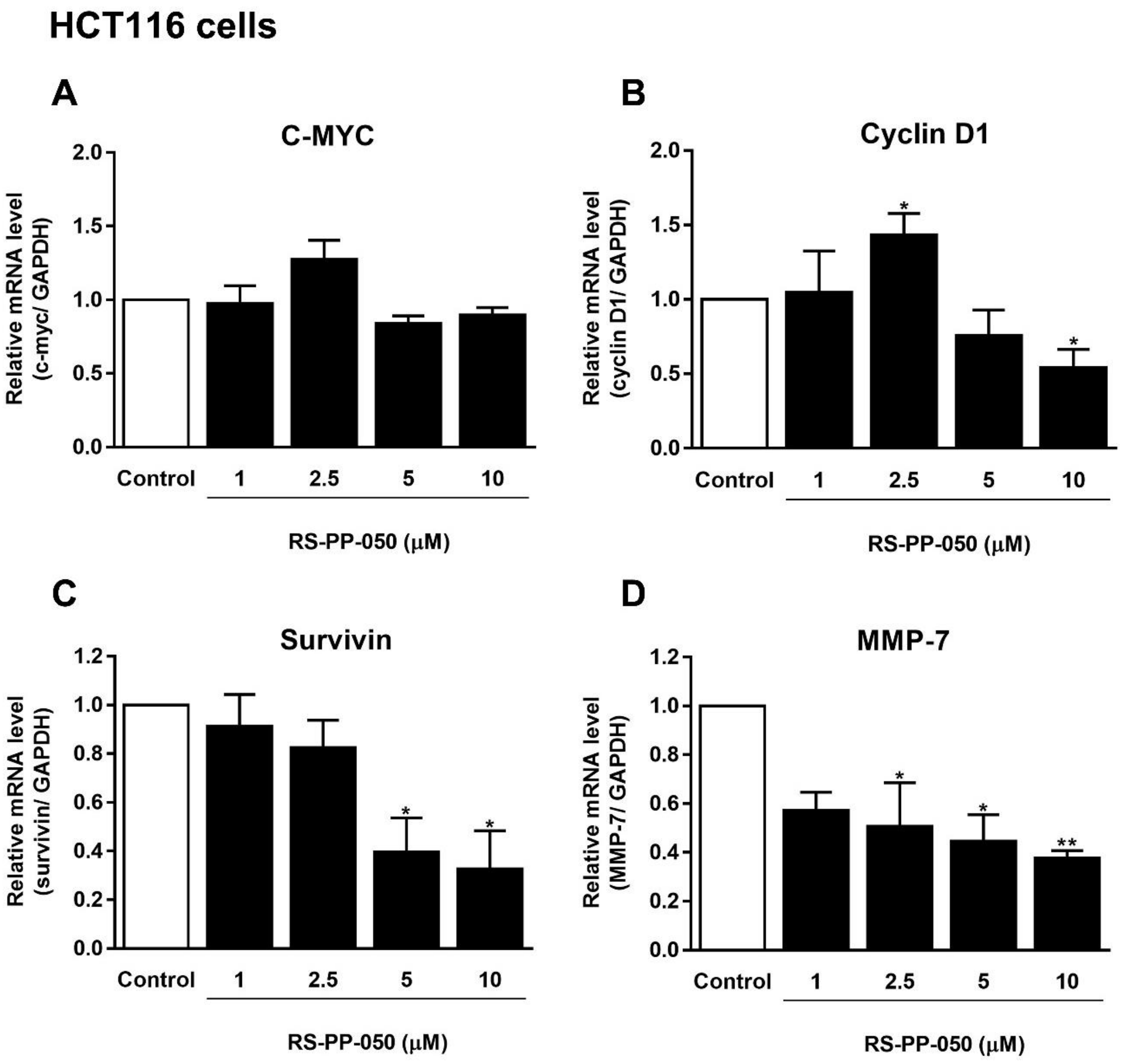

Supplement: Supplementary file 1 — Supplementary Information [file 41598_2018_26278_MOESM1_ESM.pdf]
